# Emergence and spread of a mupirocin-resistant variant of the European epidemic fusidic acid-resistant impetigo clone of *Staphylococcus aureus*, Belgium, 2013 to 2023

**DOI:** 10.2807/1560-7917.ES.2024.29.19.2300668

**Published:** 2024-05-09

**Authors:** Nicolas Yin, Charlotte Michel, Nadia Makki, Ariane Deplano, Alisha Milis, Benoit Prevost, Veronique Yvette Miendje-Deyi, Marie Hallin, Delphine Martiny

**Affiliations:** 1 National reference centre for *Staphylococcus aureus* and other species, Department of microbiology, Laboratoire Hospitalier Universitaire de Bruxelles – Universitair Laboratorium Brussel (LHUB-ULB), Université libre de Bruxelles, Brussels, Belgium; 2 Department of microbiology, Algemeen Medisch Laboratorium (AML), Antwerp, Belgium; 3 Department of microbiology, LHUB-ULB, Université libre de Bruxelles, Brussels, Belgium; 4 Centre for environmental health and occupational health, Public health school, Université libre de Bruxelles, Brussels, Belgium; 5 European Plotkin institute for vaccinology (EPIV), Faculty of medicine, Université libre de Bruxelles, Brussels, Belgium; 6 Faculty of medicine and pharmacy, Université de Mons, Mons, Belgium; * These authors contributed equally to the work and share the last authorship.

**Keywords:** Staphylococcus aureus, Impetigo, Child, Mupirocin, Fusidic Acid, Virulence, Exfoliatins, Belgium, Whole Genome Sequencing

## Abstract

**Background:**

Antimicrobial resistance to mupirocin and fusidic acid, which are used for treatment of skin infections caused by *Staphylococcus aureus,* is of concern.

**Aim:**

To investigate resistance to fusidic acid and mupirocin in meticillin-susceptible *S. aureus* (MSSA) from community-acquired skin and soft tissue infections (SSTIs) in Belgium.

**Methods:**

We collected 2013–2023 data on fusidic acid and mupirocin resistance in SSTI-associated MSSA from two large Belgian laboratories. Resistant MSSA isolates sent to the Belgian *Staphylococci* Reference Centre were *spa*-typed and analysed for the presence of the *eta* and *etb* virulence genes and the *mupA* resistance gene. In addition, we whole genome sequenced MSSA isolates collected between October 2021 and September 2023.

**Results:**

Mupirocin resistance increased between 2013 and 2023 from 0.5-1.5% to 1.7-5.6%. Between 2018 and 2023, 91.4% (64/70) of mupirocin-resistant isolates were co-resistant to fusidic acid. By September 2023, between 8.9% (15/168) and 10.1% (11/109) of children isolates from the two laboratories were co-resistant. Of the 33 sequenced isolates, 29 were sequence type 121, clonal and more distantly related to the European epidemic fusidic acid-resistant impetigo clone (EEFIC) observed in Belgium in 2020. These isolates carried the *mupA* and *fusB* genes conferring resistance to mupirocin and fusidic acid, respectively, and the *eta* and *etb* virulence genes.

**Conclusion:**

We highlight the spread of a mupirocin-resistant EEFIC in children, with a seasonal trend for the third quarter of the year. This is of concern because this variant is resistant to the two main topical antibiotics used to treat impetigo in Belgium.

**Key public health message**

**What did you want to address in this study and why?**

Bacterial skin infections, like impetigo, are often caused by *Staphylococcus aureus* and treated with antibiotic creams, either with mupirocin or fusidic acid. We wanted to investigate resistance to mupirocin and fusidic acid in *Staphylococcus aureus* involved in skin infections, particularly impetigo, in Belgium.

**What have we learnt from this study?**

Resistance to mupirocin and fusidic acid in *Staphylococcus aureus* from skin infections has increased in recent years and is linked to the emergence of a single bacterial clone with genes that confer both antibiotic resistance and increased ability to cause disease.

**What are the implications of your findings for public health?**

Treatment recommendations for impetigo and other skin infections may be reconsidered. Surveillance of skin infections should be undertaken to monitor resistance in *Staphylococcus aureus*.

## Introduction

In 2007, the *Staphylococcus aureus* European epidemic fusidic acid-resistant impetigo clone (EEFIC) was first described after an increase in fusidic acid resistance among meticillin-susceptible *S. aureus* (MSSA) had been observed in several northern European countries since ca 2000 [1]. A decade later, EEFIC incidence was considered declining in these northern European countries [2,3], while epidemiological data are lacking for Belgium. However, in a recent study, the existence and persistence of EEFIC in Belgium was shown, particularly in childhood impetigo, with a seasonal peak in late summer [4]. The spread of this clone in Belgium is of concern because topical fusidic acid is one of the recommended first-line treatments for localised impetigo in the country [5], as well as in several other European countries [6]. During this study, we identified a small number of isolates with co-resistance to mupirocin. Mupirocin-resistant EEFICs were described in Greece a few years earlier [7]. However, in contrast to the Belgian study, 80.7% of the isolates in the Greek study carried the *lukS*/*lukF* genes encoding Panton-Valentine leucocidin toxin (PVL) in addition to the *eta* and/or *etb* genes. Topical mupirocin is commonly used to treat impetigo. In France, mupirocin is recommended for localised forms of impetigo [8], and in Belgium for cases with meticillin-resistant *S. aureus* [5].

The Belgian National Reference Centre for *Staphylococcus aureus* and other species (NRC) at the Brussels University Hospital Laboratory (LHUB-ULB) routinely analyses isolates voluntarily submitted by clinical laboratories or from surveillances that may be requested by national or regional health authorities. Voluntarily submitted isolates usually have a particular antimicrobial resistance phenotype or are associated with an unusual clinical presentation, such as recurrent or severe infections or are for investigation of clusters. The request form includes information about the time of sampling, the type of sample and infection, whether a cluster was suspected or the patient had recently travelled. In summer of 2023, the NRC received an unusually high number of MSSA isolates co-resistant to fusidic acid and mupirocin and associated with skin and soft tissue infections (SSTIs). These isolates did not seem epidemiologically related, as they originated from different clinical laboratories in Flanders and Brussels. Therefore, we performed a retrospective analysis of the evolution of resistance to fusidic acid and mupirocin among community-associated MSSA isolates. We also reviewed the genetic characteristics of mupirocin- and fusidic acid-associated community-onset MSSA reported to the NRC during the same period.

## Methods

### Fusidic acid and mupirocin resistance in meticillin-susceptible *Staphylococcus aureus* from skin and soft tissue swabs

We analysed demographic and antimicrobial resistance data of MSSA isolates from SSTI swabs of outpatients between October 2013 and September 2023 from two large clinical laboratories in Belgium. In Brussels, LHUB-ULB is a clinical laboratory serving five university hospitals with a total capacity of ca 3,000 beds and a network of general practitioners in the Brussels region covering a service area of 700,000 inhabitants. In Antwerp, Algemeen Medisch Laboratorium (AML) is a private clinical laboratory belonging to the Sonic Healthcare group covering a large network of general practitioners and sampling sites in the Flanders region.

We collected data on sampling date, patient age at sampling, resistance to fusidic acid and resistance to mupirocin, as determined by Vitek 2 (bioMérieux, Marcy l'Étoile, France) according to the European Committee on Antimicrobial Susceptibility Testing (EUCAST) breakpoints [9]. Resistance to fusidic acid and mupirocin was studied over a 1-year period from October of a given year to September of the following year. As impetigo is more common in children and at the end of summer [10], a separate analysis was performed for children (< 15 years) and during the third quarter (Q3) of each year (July–September).

### Molecular analysis of fusidic acid and mupirocin co-resistant SSTI-associated meticillin-susceptible *Staphylococcus aureus*

We included MSSA isolates from skin lesions collected between October 2013 and September 2023 and sent to the NRC for detection of virulence genes. A retrospective analysis was performed on co-resistance to fusidic acid and mupirocin, as determined by disk diffusion according to EUCAST guidelines [9]. At the NRC, resistance to mupirocin was confirmed by end-point PCR detection of the *mupA* gene [11] and detection of exfoliatin genes *eta* and *etb* by end-point PCR [12]. Isolates for which at least one exfoliatin gene was detected were further analysed by *spa*-typing by sequencing the polymorphic X region of the protein A gene [13].

Isolates received between October 2021 and September 2023 were whole genome sequenced. Extraction of DNA was performed using EZ1 and 2 Virus Mini Kit v2.0 (Qiagen, Hilden, Germany) and the EZ2 Connect MDx instrument (Qiagen). Genomic DNA was enzymatically fragmented according to the manufacturer's instructions and modified to generate an Illumina-compatible DNA library using NEBNext Ultra II FS DNA Library Prep Kit for Illumina (Illumina Inc., San Diego, the United States (US)). The final libraries were qualified using an AATI Fragment Analyser (Agilent Technologies Inc., Santa Clara, US) with DNF-474 High Sensitivity NGS Fragment Analysis Kit and quantified using a Qubit 2.0 with Qubit dsDNA HS Assay Kit (Life Technologies, Carlsbad, US). After equimolar pooling, the libraries were sequenced using a NovaSeq 6000 machine (Illumina) with NovaSeq 6000 SP Reagent Kit v1.5 (300 cycles) in 2 × 150 base pairs (bp) paired mode. An average coverage of 100 × was targeted. De novo assembly was performed using the SPAdes algorithm [14]. The genome assemblies were deposited at the National Center for Biotechnology Information (NCBI) under BioProject accession number PRJNA1041362. Resistome, virulome, multilocus sequence typing (MLST), whole genome MLST (wgMLST) and *spa* type determination were performed using the BioNumerics 8.1 (bioMérieux) *S. aureus* genotyping plugin v1.1 (database *S. aureus* Virulence KB 2022.12.05 and database *S. aureus* Resistance KB 2023.10.27), the *Spa*-typing plugin v2.23, the WGS tools plugin v1.08 and MLST for the WGS plugin v1.0. A wgMLST cluster analysis was performed using the categorical distance and UPGMA (unweighted pair group method with arithmetic mean) algorithms with BioNumerics. Thirty genome assemblies of *S. aureus* clonal complex (CC) 121 strains downloaded from the NCBI GenBank database and selected from a previous study by Zhou et al. [15] to include isolates carrying *eta* and/or *etb* and to cover different countries and different *spa* types and the genome of a mupirocin-susceptible EEFIC from a previous surveillance in Belgium [4] were included as reference genomes. The list of selected isolates is provided in Supplementary Table S1.

## Results

### Fusidic acid and mupirocin resistance of meticillin-susceptible *Staphylococcus aureus* isolated from outpatient skin and soft tissue swabs

**Figure 1:**
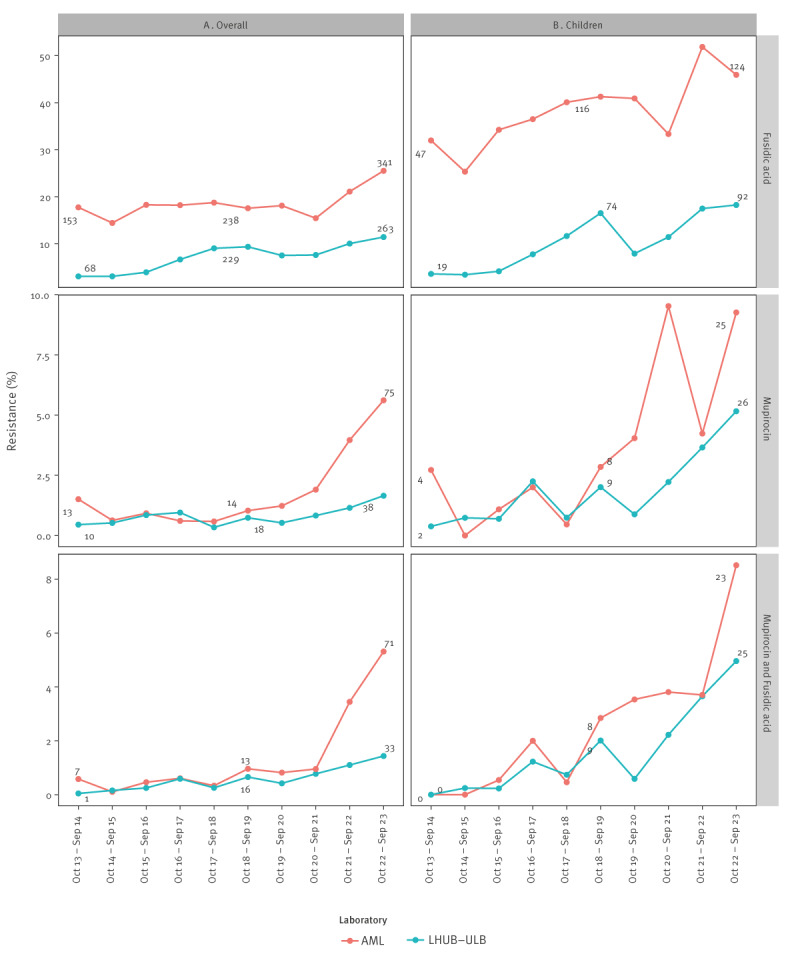
Resistance to fusidic acid and mupirocin in meticillin-susceptible *Staphylococcus aureus* isolates from skin and soft tissue infections, Belgium, October 2013–September 2023 (n = 32,503)

AML: Algemeen Medisch Laboratorium, Antwerpen; LHUB-ULB: National reference centre for Staphylococcus aureus and other species, Brussels.

Between October 2013 and September 2023, 21,232 MSSA were isolated by culture from SSTI swabs at LHUB-ULB, including 4,389 isolates from children. At AML, 11,271 SSTI-related MSSA strains were isolated, including 1,941 from children. Overall, at LHUB-ULB, resistance to fusidic acid increased from 3.1% (68/2,211) October 2013–September 2014 to 11.4% (263/2,300) October 2022–September 2023. At AML, resistance to fusidic acid increased from 17.7% (153/862) October 2013–September 2014 to 25.5% (341/1,336) October 2022–September 2023 (Figure 1). On both sites, a slight decrease was observed during the coronavirus disease 2019 (COVID-19) pandemic, 2019–2020 for LHUB-ULB and 2020–2021 for AML. During the same periods, resistance to mupirocin increased from 0.5% (10/2,211) to 1.7% (38/2,300) at LHUB-ULB and from 1.5% (13/862) to 5.6% (75/1,336) at AML. Similarly, co-resistance to fusidic acid and mupirocin increased from 0.05% (1/2,211) to 1.4% (33/2,300) at LHUB-ULB and from 0.8% (7/862) to 5.3% (71/1,336) at AML.

The number of resistant isolates per period is indicated on the graphs for the first period, the last period and September 2018–October 2019.

Similar trends, but at higher rates, were observed for children. During the study period, fusidic acid resistance increased at LHUB-ULB from 3.6% (19/527) to 18.3% (92/504) and from 32.0% (47/147) to 45.9% (124/270) at AML. Resistance to mupirocin increased at LHUB-ULB from 0.4% (2/527) to 5.2% (26/504) and at AML from 2.7% (4/147) to 9.3% (25/270). Co-resistance to fusidic acid and mupirocin increased in LHUB-ULB from 0% (0/527) to 5.0% (25/504) and at AML from 0% (0/147) to 8.5% (23/270).

**Figure 2:**
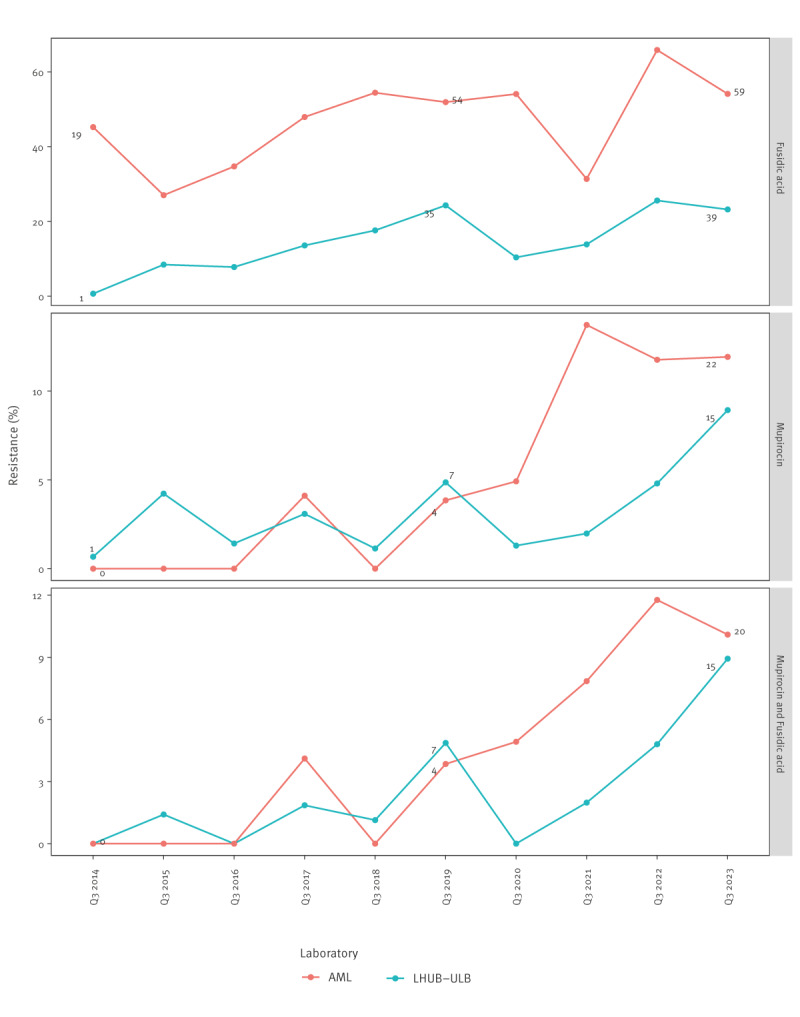
Resistance to fusidic acid and mupirocin in meticillin-susceptible *Staphylococcus aureus* isolates from skin and soft tissue infections in children, Belgium, July–September 2014–2023 (n = 2,038)

AML: Algemeen Medisch Laboratorium, Antwerpen; LHUB-ULB: National reference centre for Staphylococcus aureus and other species, Brussels; Q3: July–September.

In isolates from children in Q3 of each year, resistance to fusidic acid increased at LHUB-ULB from 0.7% (1/149) in 2014 to 23.2% (39/168) in 2023 and at AML from 45.2% (19/42) in 2014 to 54.1% (59/109) in 2023 (Figure 2). Resistance to mupirocin increased at LHUB-ULB from 0.7% (1/149) in 2014 to 8.9% (15/168) in 2023 and at AML from 0% (0/42) in 2014 to 11.9% (13/109) in 2023. Co-resistance to fusidic acid and mupirocin increased at LHUB-ULB from 0% (0/149) to 8.9% (15/168) and at AML from 0% (0/42) to 10.1% (11/109). Between October 2018 and September 2023, 91.4% (64/70) of the mupirocin-resistant MSSA strains were co-resistant to fusidic acid. The raw data are shown in Supplementary Table S2.

The number of resistant isolates per period is indicated on the graphs for the first period, the last period and Q3 2019.

### Genomic study of fusidic acid and mupirocin coresistant SSTI-associated MSSA

Between October 2013 and September 2023, the NRC received 58 MSSA isolates co-resistant to mupirocin and fusidic acid. These isolates from SSTIs were submitted by 29 laboratories across Belgium (Table). No suspicion of a cluster or recent travel history was reported on any of the accompanying request forms. No laboratory sent more than three isolates per period. Of the 58 isolates, 37 originated from Flanders, 12 from the Brussels region and 9 from Wallonia. The number of isolates received increased from no isolates between 2013 and 2014 to 19 between 2022 and 2023. Most (n = 40) of the 58 isolates were from children, 51 of 57 tested isolates were positive for *eta* and 47 of 57 for *etb*. All 39 isolates tested for *mupA* were positive. Of the 54 *spa*-typings performed, 35 were t1994 and 5 had closely related *spa* types (t162, t2391, t21368), all of which were closely related to t171, which was associated with the original description of EEFIC [1]. Furthermore, all these 40 isolates carried *eta* and *etb*. Therefore, since October 2017, 40 of the 51 isolates co-resistant to fusidic acid and mupirocin from SSTIs in Belgium have been similar to EEFIC, but in addition to their exfoliatins and fusidic acid resistance, they also carry the *mupA* gene, which confers resistance to mupirocin.

**Table:** Characteristics of fusidic acid- and mupirocin-resistant meticillin-susceptible *Staphylococcus aureus* isolates from skin and soft tissue infections, Belgium, October 2014–September 2023 (n = 58)

Date	Isolates	Laboratories	Patients age < 15 years	*eta*		*etb*		*mupA*		CC121-related *spa* type	
				Tested	Positive	Tested	Positive	Tested	Positive	Tested	Identified
Oct 2014–Sep 2015	1	1	0	1	0	1	0	1	1	1	0
Oct 2015–Sep 2016	1	1	0	1	0	1	0	1	1	1	0
Oct 2016– Sep 2017	1	1	0	1	0	1	0	1	1	1	0
Oct 2017–Sep 2018	7	3	7	7	7	7	7	7	7	7	6
Oct 2018–Sep 2019	6	5	3	6	5	6	5	5	5	6	5
Oct 2019–Sep 2020	4	3	4	4	4	4	3	1	1	4	3
Oct 2020–Sep 2021	5	5	4	5	5	5	4	3	3	5	4
Oct 2021–Sep 2022	14	9	8	14	13	14	12	5	5	13	9
Oct 2022–Sep 2023	19	14	14	18	17	18	16	15	15	16	13
Total	58	29	40	57	51	57	47	39	39	54	40

CC: *Staphylococcus aureus* clonal complex; *eta*: detection of exfoliatin a gene; *etb*: detection of exfoliatin b gene; *mupA*: detection of mupA gene conferring resistance to mupirocin.

**Figure 3:**
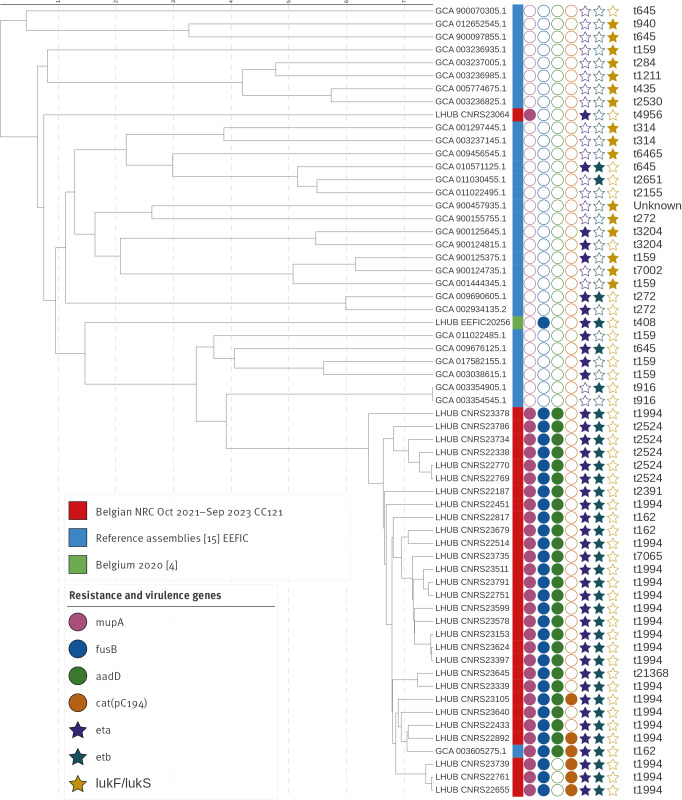
*spa* types and dendrogram of whole genome multilocus sequence typing results of meticillin-susceptible *Staphylococcus aureus* sequence type 121 reference genomes (n = 30), sequence type 121 isolates from Belgium, October 2021–September 2023 (n = 30) and sequence type 123 isolate from Belgium, 2020 (n = 1)

Whole genome sequencing was performed on 33 isolates received at the NRC between October 2021 and September 2023. These isolates came from 19 different laboratories. Twenty isolates were from Flanders, 7 from Wallonia and 6 from the Brussels region. All isolates carried *mupA,* with 30 of 33 ST121; hence, these strains are CC121 strains. Of these 30, 28 carried *eta* and all carried *etb*. None of them carried *lukS/lukF*. Twenty-nine carried the acquired gene *fusB*, while one isolate had the L461K-mutated chromosomal *fusA*, conferring resistance to fusidic acid. In addition, of 30 isolates, 26 carried the *aadD* gene, which confers resistance to amikacin and six the *cat(pC194)* gene, which confers resistance to chloramphenicol. The remaining three isolates were negative for *etb*, two were ST45 and negative for *eta* (*spa* type t550) and one was ST15 (*spa* type t084) and harboured *etb*. The wgMLST analysis showed that the 29 ST121 isolates carrying *fusB* were likely clonal. In addition, the location of *fusB* in these isolates was away from *groEL*, contrary to the original description of EEFIC [1], but the isolates carried the gene encoding epidermal differentiation inhibitor C (EDIN-C) close to *etb*. These isolates clustered with the reference genome of an MSSA isolated in Greece in 2018 (GenBank accession GCA_003605275.1). This MSSA carried *mupA*, *fusB*, *aadD*, *eta* and *etb*. These isolates were more closely related to each other than to the ST123 EEFIC observed in Belgium in 2020, while the ST121 MSSA with the *fusA* mutation is even more distantly related (Figure 3). Additional data are shown in Supplementary Table S3.

*aadD*: gene conferring resistance to amikacin; *cat(pC194)*: gene conferring resistance to chloramphenicol; *eta*: exfoliatin a gene; *etb*: exfoliatin b gene; *lukS/lukF*: genes encoding Panton-Valentine leucocidin toxin (PVL); MSSA: methicillin-susceptible Staphylococcus aureus; *mupA*: gene conferring resistance to mupirocin; ST: sequence type.

Red squares represent ST121 MSSA isolates from Belgium October 2021–September 2023; blue squares represent reference genomes of ST121 MSSA (Zhou et al.) [15] and green square represents ST123 MSSA belonging to the European epidemic fusidic acid-resistant impetigo clone from Belgium, 2020 (Deplano et al.) [4].

Filled circles indicate the detection of resistance genes and filled stars indicate the detection of virulence genes.

## Discussion

Overall, mupirocin resistance seems to have increased since October 2019. It remained at low level (below 2%) in isolates from LHUB-ULB, whereas it reached 5.6% at AML. Focusing on the paediatric population, a worrying trend is observed for years 2018–2023: the proportion of mupirocin resistance reached more than 5% for the period October 2022-September 2023 and was almost systematically associated with resistance to fusidic acid (64/70, 91.4%). Focusing on children during Q3, the impetigo season [10], revealed an even more alarming trend, with mupirocin resistance rates ranging from 8.9% to 11.9% and an almost systematic association with fusidic acid resistance (26/28). Based on the analysis of the isolates received by the NRC since October 2017, 40 of the 51 mupirocin and fusidic acid co-resistant isolates were confirmed related to EEFIC. Whole genome sequencing of isolates collected between October 2021 and September 2023 confirmed this concern, with 29 of 33 clonal isolates sharing the following genetic characteristics: ST121, resistance to fusidic acid conferred by *fusB*, resistance to mupirocin conferred by *mupA* and the presence of the virulence genes *eta* and *etb*. In a previous study on subpopulations of *S. aureus* CC121, isolates sampled from superficial infections (including staphylococcal scalded skin syndrome, bullous impetigo, exfoliative dermatitis, and conjunctivitis) clustered in the same clade, including EEFIC [16]. Interestingly, wgMLST cluster analysis showed that our isolates are closely related to the reference genome of an MSSA isolated in Greece, which shares the same characteristics, while the other reference genomes from SSTI-related isolates are more distantly related. However, in contrast to a previous study in Greece describing the emergence of a similar ST121 clone [7], none of them carried the *lukS*/*lukF* genes. Notably, one of these 33 isolates was ST15 with a t084 *spa* type and had *etb*. Isolates of ST15 carrying exfoliatin genes have been observed quite frequently in staphylococcal scalded skin syndrome in France [17].

Treatment of impetigo is often based on the use of topical antibiotics for localised forms, such as fusidic acid cream in Belgium and the Netherlands [5,6] or mupirocin in France [8]. This option is preferred for localised disease because it has been at least as effective as oral treatment, with fewer side effects [18,19]. Therefore, the emergence and spread of an MSSA clone co-resistant to these two antibiotics is a cause for concern. Other possible topical treatments are retapamulin [18,20] and ozenoxacin [21]. However, in the European Union, marketing authorisation for retapamulin was withdrawn by the European Commission on 25 February 2017 at the request of the manufacturer. At the time of manuscript writing, ozenoxacin was only available in Italy, Portugal and Spain [22]. Interestingly, the emerging clone described in the present study frequently carried *aadD* and *cat(pC194)*, which confer resistance to amikacin and chloramphenicol, respectively. Although these antibiotics are not used in Belgium, they may be used as topical treatments in other countries. Acquisition of these resistance genes is likely a selective advantage for strains with a propensity for superficial skin infections. On the other hand, the efficacy of disinfection as sole treatment for impetigo seems to be inferior to that of topical antibiotics [18], even though recommendations in the United Kingdom favour the single use of antiseptics [23]. However, the development of resistance to topical antibiotics may change this consideration, and further studies are needed to determine the best interventions for localised impetigo.

Impetigo is known to affect children in summer [10]. Indeed, in a previous survey focused on *S. aureus*-related SSTIs, we showed that EEFIC was more prevalent in children in late summer [4]. At that time, resistance to mupirocin was anecdotal. However, one of the limitations of this previous study was that it was conducted during the summer immediately after onset of the COVID-19 crisis. The social distancing and restrictions that gradually came into force during this period, combined with the workload of the clinical laboratories during the study period, certainly introduced biases. The end of the COVID-19-related restrictions and the increase in mupirocin-resistant strains arriving at the NRC led us to perform the present study using immediately available retrospective laboratory data. As isolates are sent to the NRC by clinical laboratories on a voluntary basis, the NRC data may not always reflect the true epidemiological situation. To determine the accuracy of the emergence of a new clone, we collected epidemiological data from two large laboratories in Belgium (LHUB-ULB and AML). In a previous study focusing on respiratory viruses, LHUB-ULB was demonstrated to act as a sensor for the whole country due to the large number of analyses and its central location within Belgium [24]. Inclusion of AML allowed the addition of data from a different type of laboratory as LHUB-ULB is more of a hospital laboratory, whereas AML, located in Flanders, mainly collects samples directly from general practitioners and outpatient sampling centres. Collecting data from a laboratory in the third region of Belgium (Wallonia) would have covered the third region of the country. However, we did not succeed in finding another laboratory with sufficient data to join the study. Setting up an actual prospective surveillance would have been more accurate but might have delayed communication of these results. Nevertheless, using data from two laboratories only has limitations. The current data are not fully representative of the entire country. Furthermore, the proportion of skin lesions that are sampled before treatment is unknown, and the total number of skin samples sent to laboratories per year is not known, as these data were not collected by health insurance providers. This representativeness bias is limited by the overall number of isolates included (32,503 MSSA over 10 years) but may lead to some imprecision when focusing on a specific population during a specific time period, as we did in this study for children during the third quarter of the year. The epidemiological differences observed between AML and LHUB-ULB could be due to regional differences and/or differences between the patients sampled (case mix). The impact of the latter is likely mitigated by the fact that we specifically selected samples from outpatients. Nevertheless, AML collects samples from the Federal Agency for reception of asylum seekers (FEDASIL), and identification of epidemiological clusters at the level of the clinical laboratory is not easy because the context is rarely provided by the prescribers for outpatients on the forms following the specimens. Regional epidemiological differences may exist. For example, in the most recent surveillance study on EEFIC in Belgium [4], all EEFICs were from Flanders, and almost half of them (47.4%) were in the province of Antwerp, where AML is located. Between October 2013 and September 2023, most (37/58) MSSA strains co-resistant to mupirocin and fusidic acid analysed by the NRC in this study were from Flanders, followed by Brussels and Wallonia. Nevertheless, it is also possible that laboratories and practitioners in Flanders are more aware of the current epidemiological situation due to the EEFIC clusters in 2018 that led to our previous surveillance; in contrast, these clusters were not described in Wallonia.

Although very common, impetigo is likely an understudied pathology [25]. Indeed, there is currently no organised surveillance of impetigo in Belgium. Consequently, epidemiological data rely on laboratory data, as in this study. At the laboratory level, clinical information on the type of infection is often lacking. We attempted to overcome these problems in our study by including skin samples from outpatients only and by focusing on the more likely target population for impetigo: children sampled in late summer (Q3). Additionally, uncomplicated impetigo may not be sampled. This may lead to bias towards complicated skin infections that should not be treated with topical antibiotics. Nevertheless, this study clearly demonstrated the emergence of a clone combining virulence and resistance. Periodic surveillance is needed to better monitor the true epidemiology of impetigo and adapt treatment recommendations accordingly. In addition, this surveillance may provide useful information for developing intervention trials for impetigo patients.

## Conclusion

The present study highlights the emergence and spread of a clonal ST121 mupirocin-resistant clone diverging from EEFIC (M-EEFIC), which has acquired the *mupA* gene in addition to its virulence (*eta* and *etb*) and resistance (*fusB*) genes. This clone could have a selective advantage in Belgium and similar European countries where there is no other alternative topical treatment. The emergence of this new clone, combined with the overall high prevalence of fusidic acid resistance, should lead to reconsideration of the recommendations for first-line treatment of impetigo in Belgium and introduction of active surveillance of impetigo to better assess the situation.

## Supplementary Data

165000 Supplementary Material

## References

[1] O’Neill AJ Larsen AR Skov R Henriksen AS Chopra I . Characterization of the epidemic European fusidic acid-resistant impetigo clone of Staphylococcus aureus. J Clin Microbiol. 2007;45(5):1505-10. 10.1128/JCM.01984-06 17344365 PMC1865894

[2] Rørtveit S Skutlaberg DH Langeland N Rortveit G . The decline of the impetigo epidemic caused by the epidemic European fusidic acid-resistant impetigo clone: an 11.5-year population-based incidence study from a community in Western Norway. Scand J Infect Dis. 2014;46(12):832-7. 10.3109/00365548.2014.947317 25229166 PMC4266085

[3] Dalager-Pedersen M Søgaard M Schønheyder HC . Staphylococcus aureus skin and soft tissue infections in primary healthcare in Denmark: a 12-year population-based study. Eur J Clin Microbiol Infect Dis. 2011;30(8):951-6. 10.1007/s10096-011-1179-0 21279531

[4] Deplano A Hallin M Bustos Sierra N Michel C Prevost B Martiny D Persistence of the Staphylococcus aureus epidemic European fusidic acid-resistant impetigo clone (EEFIC) in Belgium. J Antimicrob Chemother. 2023;78(8):2061-5. 10.1093/jac/dkad204 37358399 PMC10393872

[5] Belgian Antibiotic Policy Coordination Commission (BAPCOC). Guide belge de traitement anti-infectieux en pratique ambulatoire/Belgische gids voor anti-infectieuze behandeling in de ambulante praktijk. [Belgian guide for treatment of infections in outpatient practice]. Brussels: BAPCOC; Nov 2022. French/Dutch. Available from: https://organesdeconcertation.sante.belgique.be/fr/documents/guide-belge-de-traitement-anti-infectieux-en-pratique-ambulatoire-2022

[6] Nederlands Huisartsen Genootschap (NHG). Bacteriële huidinfecties (M68). [Bacterial skin infections]. Utrecht: NHG; May 2019. Dutch. Available from: https://richtlijnen.nhg.org/files/pdf/61_Bacteri%C3%ABle%20huidinfecties_mei-2019.pdf

[7] Doudoulakakis A Spiliopoulou I Spyridis N Giormezis N Kopsidas J Militsopoulou M Emergence of a Staphylococcus aureus clone resistant to mupirocin and fusidic acid carrying exotoxin genes and causing mainly skin infections. J Clin Microbiol. 2017;55(8):2529-37. 10.1128/JCM.00406-17 28592549 PMC5527431

[8] Haute autorité de santé (HAS). Prise en charge des infections cutanées bactériennes courantes. [Management of common bacterial skin infections]. Saint-Denis: HAS; Feb 2019. French. Available from: https://www.has-sante.fr/upload/docs/application/pdf/2019-04/prise_en_charge_des_infections_cutanees_bacteriennes_courantes_recommandations.pdf

[9] The European Committee on Antimicrobial Susceptibility Testing (EUCAST). Breakpoint tables for interpretation of MICs and zone diameters. Version 11.0, Jan 2023. Available from: https://www.eucast.org/fileadmin/src/media/PDFs/EUCAST_files/Disk_test_documents/2023_manuals/Manual_v_11.0_EUCAST_Disk_Test_2023.pdf

[10] Loffeld A Davies P Lewis A Moss C . Seasonal occurrence of impetigo: a retrospective 8-year review (1996-2003). Clin Exp Dermatol. 2005;30(5):512-4. 10.1111/j.1365-2230.2005.01847.x 16045681

[11] Ramsey MA Bradley SF Kauffman CA Morton TM . Identification of chromosomal location of mupA gene, encoding low-level mupirocin resistance in staphylococcal isolates. Antimicrob Agents Chemother. 1996;40(12):2820-3. 10.1128/AAC.40.12.2820 9124848 PMC163629

[12] Jarraud S Mougel C Thioulouse J Lina G Meugnier H Forey F Relationships between Staphylococcus aureus genetic background, virulence factors, agr groups (alleles), and human disease. Infect Immun. 2002;70(2):631-41. 10.1128/IAI.70.2.631-641.2002 11796592 PMC127674

[13] Hallin M Deplano A Denis O De Mendonça R De Ryck R Struelens MJ . Validation of pulsed-field gel electrophoresis and spa typing for long-term, nationwide epidemiological surveillance studies of Staphylococcus aureus infections. J Clin Microbiol. 2007;45(1):127-33. 10.1128/JCM.01866-06 17093021 PMC1828992

[14] Bankevich A Nurk S Antipov D Gurevich AA Dvorkin M Kulikov AS SPAdes: a new genome assembly algorithm and its applications to single-cell sequencing. J Comput Biol. 2012;19(5):455-77. 10.1089/cmb.2012.0021 22506599 PMC3342519

[15] Zhou W Jin Y Teng G Chen W Chen Y Luo Q Comparative analysis of genomic characteristics, virulence and fitness of community-associated *Staphylococcus aureus* ST121 clone causing fatal diseases in China and other CA-MRSA clones. Virulence. 2023;14(1):2242547. 10.1080/21505594.2023.2242547 37534993 PMC10402838

[16] Kurt K Rasigade JP Laurent F Goering RV Žemličková H Machova I Subpopulations of Staphylococcus aureus clonal complex 121 are associated with distinct clinical entities. PLoS One. 2013;8(3):e58155. 10.1371/journal.pone.0058155 23505464 PMC3591430

[17] Lamand V Dauwalder O Tristan A Casalegno JS Meugnier H Bes M Epidemiological data of staphylococcal scalded skin syndrome in France from 1997 to 2007 and microbiological characteristics of Staphylococcus aureus associated strains. Clin Microbiol Infect. 2012;18(12):E514-21. 10.1111/1469-0691.12053 23078129

[18] Koning S van der Sande R Verhagen AP van Suijlekom-Smit LW Morris AD Butler CC Interventions for impetigo. Cochrane Database Syst Rev. 2012;1(1):CD003261. 22258953

[19] George A Rubin G . A systematic review and meta-analysis of treatments for impetigo. Br J Gen Pract. 2003;53(491):480-7. 12939895 PMC1314624

[20] Yang LPH Keam SJ . Retapamulin: a review of its use in the management of impetigo and other uncomplicated superficial skin infections. Drugs. 2008;68(6):855-73. 10.2165/00003495-200868060-00008 18416589

[21] Rosen T Albareda N Rosenberg N Alonso FG Roth S Zsolt I Efficacy and safety of ozenoxacin cream for treatment of adult and pediatric patients with impetigo: a randomized clinical trial. JAMA Dermatol. 2018;154(7):806-13. 10.1001/jamadermatol.2018.1103 29898217 PMC6128489

[22] European Medicines Agency (EMA). Medicines. Amsterdam: EMA. [Accessed: 21 Feb 2024]. Available from: https://www.ema.europa.eu/en/medicines

[23] National Institute for Health and Care Excellence (NICE). Impetigo: antimicrobial prescribing. London: NICE; 26 Feb 2020. Available from: https://www.nice.org.uk/guidance/ng153

[24] Van den Wijngaert S Bossuyt N Ferns B Busson L Serrano G Wautier M Bigger and Better? Representativeness of the influenza A surveillance using one consolidated clinical microbiology laboratory data set as compared to the Belgian Sentinel Network of Laboratories. Front Public Health. 2019;7:150. 10.3389/fpubh.2019.00150 31275914 PMC6591264

[25] Gorges H Hall L Heal C . Feasibility study for a randomised controlled trial for the topical treatment of impetigo in Australian general practice. Trop Med Infect Dis. 2021;6(4):197. 10.3390/tropicalmed6040197 34842831 PMC8628881

