# Supplementary material for: Emergence and spread of a mupirocin-resistant variant of the European epidemic fusidic acid-resistant impetigo clone of Staphylococcus aureus, Belgium, 2013 to 2023
Source: Euro Surveill. 2024 May 9;29(19):2300668. doi: 10.2807/1560-7917.ES.2024.29.19.2300668 (PMC11083972; doi:10.2807/1560-7917.ES.2024.29.19.2300668)
Supplement: Supplementary Material [file 23-00668_YIN_Supplement.pdf]

Strain	GenBank/SRA accessior	Length	Year of isolatior	Country	Host	Isolation source	*spa* type
BU_G1201_t8	GCA_001297445.1	2759850	2013	Ghana	Human	Wound	t314
XQ	GCA_001444345.1	2803594	2009	China	Human	Blood	t159
G703N1B1	GCA_002934135.2	2806379	2017	South Africa	Human	Respiratory tract	t272
SAMNA5	GCA_003038615.1	2796695	2016	Lebanon	Human	Skin/soft tissue	t159
CM172	GCA_003236825.1	2762507	2014	Italy	Human	Skin/soft tissue	t2530
CM164	GCA_003236935.1	2791197	2013	Italy	Human	Skin/soft tissue	t159
CM161	GCA_003236985.1	2817926	2013	Italy	Human	Skin/soft tissue	t1211
CM160	GCA_003237005.1	2808882	2013	Italy	Human	Skin/soft tissue	t284
CM146	GCA_003237145.1	2755498	2014	Italy	Human	Skin/soft tissue	t314
27	GCA_003354545.1	2814328	1994	Germany	Human	Blood	t916
SauNA3893Lar	GCA_003605275.1	2845940	2014	Greece	Human	NA	t162
0137NA2003NA2005	GCA_005774675.1	2824683	2005	Russia	Human	Blood	t435
MRSA738	GCA_009456545.1	2798198	2016	USA	Human	Wound	t6465
SA29KEN	GCA_009690605.1	2834535	2015	Kenya	Human	NA	t272
UC487	GCA_011022495.1	2752075	2012	Latin America	Human	Blood	t2155
CV15	GCA_012652545.1	2786059	1997	Cape Verde	Human	Respiratory tract	t940
6160	GCA_017582155.1	2772609	2016	France	Human	Blood	t159
1943STDY5573617	GCA_900070305.1	2678107	1999	United Kingdom	Animal	Animal	t645
MSSA777507T	GCA_900097855.1	2823466	2013	Tanzania	Human	Wound	t645
3688STDY6124979	GCA_900124735.1	2798059	2015	Thailand	Human	Unknown sterile site	t7002
CHUV_13	GCA_900155755.1	2785685	2014	Switzerland	Human	Skin/soft tissue	t272
78	GCA_003354905.1	2853623	1994	Germany	Human	Respiratory tract	t916
UA828	GCA_009676125.1	2814267	2013	Latin America	Human	Blood	t645
5300	GCA_010571125.1	2846135	2018	Latin America	Human	Blood	t645
UCL322	GCA_011022485.1	2757608	2012	Latin America	Human	Blood	t159
UB494	GCA_011030455.1	2828401	2012	Latin America	Human	Blood	t2651
3688STDY6124962	GCA_900124815.1	2832226	2015	Thailand	Human	Unknown sterile site	t3204
3688STDY6124814	GCA_900125375.1	2842272	2015	Thailand	Human	Unknown sterile site	t159
3688STDY6124848	GCA_900125645.1	2852478	2015	Thailand	Human	Unknown sterile site	t3204
NCTC3750	GCA_900457935.1	2892877	1932	United Kingdom	Human	Skin/soft tissue	NA

Table S1. Information on ST121 isolates analysed in this study.

This supplementary material is hosted by Eurosurveillance as supporting information alongside the article "Emergence and spread of a mupirocin-resistant variant of the European epidemic fusidic acid-resistant impetigo clone of *Staphylococcus aureus* . Belgium, 2013 to 2023", on behalf of the authors, who remain responsible for the accuracy and appropriateness of the content. The same standards for ethics, copyright, attributions and permissions as for the article apply. Supplements are not edited by Eurosurveillance and the journal is not responsible for the maintenance of any links or email addresses provided therein.

Time	Data	Laboratory	MSSA	FA-R	MUP-R	FA-R/MUP-R
Oct 13 - Sep 14	Children	AML	147	47	4	0
Oct 14 - Sep 15	Children	AML	150	38	0	0
Oct 15 - Sep 16	Children	AML	184	63	2	1
Oct 16 - Sep 17	Children	AML	200	73	4	4
Oct 17 - Sep 18	Children	AML	217	87	1	1
Oct 18 - Sep 19	Children	AML	281	116	8	8
Oct 19 - Sep 20	Children	AML	198	81	8	7
Oct 20 - Sep 21	Children	AML	105	35	10	4
Oct 21 - Sep 22	Children	AML	189	98	8	7
Oct 22 - Sep 23	Children	AML	270	124	25	23
Oct 13 - Sep 14	Children	LHUB-ULB	527	19	2	0
Oct 14 - Sep 15	Children	LHUB-ULB	408	14	3	1
Oct 15 - Sep 16	Children	LHUB-ULB	433	18	3	1
Oct 16 - Sep 17	Children	LHUB-ULB	489	38	11	6
Oct 17 - Sep 18	Children	LHUB-ULB	541	63	4	4
Oct 18 - Sep 19	Children	LHUB-ULB	448	74	9	9
Oct 19 - Sep 20	Children	LHUB-ULB	341	27	3	2
Oct 20 - Sep 21	Children	LHUB-ULB	315	36	7	7
Oct 21 - Sep 22	Children	LHUB-ULB	383	67	14	14
Oct 22 - Sep 23	Children	LHUB-ULB	504	92	26	25
Oct 13 - Sep 14	Overall	AML	862	153	13	7
Oct 14 - Sep 15	Overall	AML	956	138	6	1
Oct 15 - Sep 16	Overall	AML	1083	198	10	5
Oct 16 - Sep 17	Overall	AML	1153	210	7	7
Oct 17 - Sep 18	Overall	AML	1200	225	7	4
Oct 18 - Sep 19	Overall	AML	1355	238	14	13
Oct 19 - Sep 20	Overall	AML	1220	221	15	10
Oct 20 - Sep 21	Overall	AML	945	146	18	9
Oct 21 - Sep 22	Overall	AML	1161	245	46	40
Oct 22 - Sep 23	Overall	AML	1336	341	75	71
Oct 13 - Sep 14	Overall	LHUB-ULB	2211	68	10	1
Oct 14 - Sep 15	Overall	LHUB-ULB	1913	59	10	3
Oct 15 - Sep 16	Overall	LHUB-ULB	2004	79	17	5
Oct 16 - Sep 17	Overall	LHUB-ULB	2204	147	21	13
Oct 17 - Sep 18	Overall	LHUB-ULB	2355	213	8	6
Oct 18 - Sep 19	Overall	LHUB-ULB	2445	229	18	16
Oct 19 - Sep 20	Overall	LHUB-ULB	1900	143	10	8
Oct 20 - Sep 21	Overall	LHUB-ULB	1810	138	15	14
Oct 21 - Sep 22	Overall	LHUB-ULB	2090	210	24	23
Oct 22 - Sep 23	Overall	LHUB-ULB	2300	263	38	33
Q3 2014	Q3+Children	AML	42	19	0	0
Q3 2015	Q3+Children	AML	37	10	0	0
Q3 2016	Q3+Children	AML	72	25	0	0
Q3 2017	Q3+Children	AML	73	35	3	3
Q3 2018	Q3+Children	AML	90	49	0	0
Q3 2019	Q3+Children	AML	104	54	4	4
Q3 2020	Q3+Children	AML	61	33	3	3
Q3 2021	Q3+Children	AML	51	16	7	4
Q3 2022	Q3+Children	AML	85	56	10	10
Q3 2023	Q3+Children	AML	109	59	13	11
Q3 2014	Q3+Children	LHUB-ULB	149	1	1	0
Q3 2015	Q3+Children	LHUB-ULB	71	6	3	1
Q3 2016	Q3+Children	LHUB-ULB	141	11	2	0
Q3 2017	Q3+Children	LHUB-ULB	162	22	5	3
Q3 2018	Q3+Children	LHUB-ULB	176	31	2	2
Q3 2019	Q3+Children	LHUB-ULB	144	35	7	7
Q3 2020	Q3+Children	LHUB-ULB	77	8	1	0
Q3 2021	Q3+Children	LHUB-ULB	101	14	2	2
Q3 2022	Q3+Children	LHUB-ULB	125	32	6	6
Q3 2023	Q3+Children	LHUB-ULB	168	39	15	15

MSSA: methicillin-susceptible *Staphylococcus aureus* FA: Fusidic Acid, MUP: Mupirocin, -R: resistant, Q3: 3rd trimester, Sep: September, Oct: October

Table S2: Number of skin swabs positive for methicillin-susceptible *Staphylococcus aureus* and their resistance to mupirocin and fusidic acid in 2 Belgian

This supplementary material is hosted by Eurosurveillance as supporting information alongside the article "Emergence and spread of a mupirocin-resistant variant of the European epidemic fusidic acid-resistant impetigo clone of *Staphylococcus aureus* . Belgium, 2013 to 2023", on behalf of the authors, who remain responsible for the accuracy and appropriateness of the content. The same standards for ethics, copyright, attributions and permissions as for the article apply. Supplements are not edited by Eurosurveillance and the journal is not responsible for the maintenance of any links or email addresses provided therein.

Isolate	MLST	spa-Type	wgMLST	Resistance genes										Resistance mutations					Virulence genes			
				mupA	fusB	tet(K)	cat(pC194)	cat(pC221)	erm(C)	erm(A)	dfrG	blaZ	aadD	fusA_pH457	fusA_pL461K	grlA_pI45M	grlB_pE422D	mupA_pS570	etb	eta	lukS-PV	lukF-PV
LHUB_CNRS22187	121	t2391	publicST121	1	1	0	0	0	0	0	0	1	1	0	0	0	1	0	1	1	0	0
LHUB_CNRS22433	121	t1994	publicST121	1	1	0	0	0	0	0	0	1	1	0	0	0	1	0	1	1	0	0
LHUB_CNRS22338	121	t2524	publicST121	1	1	0	0	0	0	0	0	1	1	0	0	0	1	0	1	1	0	0
LHUB_CNRS22451	121	t1994	publicST121	1	1	0	0	0	0	0	0	1	1	0	0	0	1	0	1	1	0	0
LHUB_CNRS22514	121	t1994	publicST121	1	1	0	0	0	0	0	0	1	1	0	0	0	1	0	1	1	0	0
LHUB_CNRS22539	45	t550	publicST45	1	0	0	0	0	0	0	0	1	0	0	1	1	1	1	0	0	0	0
LHUB_CNRS22655	121	t1994	publicST121	1	1	0	1	0	0	0	0	1	0	0	0	0	1	0	1	1	0	0
LHUB_CNRS22751	121	t1994	publicST121	1	1	0	0	0	0	0	0	1	1	0	0	0	1	0	1	1	0	0
LHUB_CNRS22757	15	t084	publicST15	1	1	0	0	0	0	0	0	1	0	0	0	0	0	0	0	1	0	0
LHUB_CNRS22761	121	t1994	publicST121	1	1	0	1	0	0	0	0	1	0	0	0	0	1	0	1	1	0	0
LHUB_CNRS22769	121	t2524	publicST121	1	1	0	0	0	0	0	0	1	1	0	0	0	1	0	1	1	0	0
LHUB_CNRS22770	121	t2524	publicST121	1	1	0	0	0	0	0	0	1	1	0	0	0	1	0	1	1	0	0
LHUB_CNRS22817	121	t162	publicST121	1	1	0	0	0	0	0	0	1	1	0	0	0	1	0	1	1	0	0
LHUB_CNRS22892	121	t1994	publicST121	1	1	0	1	0	0	0	0	1	1	1	0	0	1	0	1	1	0	0
LHUB_CNRS23064	121	t4956	publicST121	1	0	0	0	0	1	0	0	1	0	0	1	0	1	0	0	1	0	0
LHUB_CNRS23105	121	t1994	publicST121	1	1	0	1	0	0	0	0	1	1	0	0	0	1	0	1	1	0	0
LHUB_CNRS23153	121	t1994	publicST121	1	1	0	0	0	0	0	0	1	1	0	0	0	1	0	1	1	0	0
LHUB_CNRS23339	121	t1994	publicST121	1	1	0	0	0	0	0	0	1	1	0	0	0	1	0	1	1	0	0
LHUB_CNRS23378	121	t1994	publicST121	1	1	0	0	0	0	0	0	1	1	0	0	0	1	0	1	1	0	0
LHUB_CNRS23397	121	t1994	publicST121	1	1	0	0	0	0	0	0	1	1	0	0	0	1	0	1	1	0	0
LHUB_CNRS23511	121	t1994	publicST121	1	1	0	0	0	0	0	0	1	1	0	0	0	1	0	1	1	0	0
LHUB_CNRS23578	121	t1994	publicST121	1	1	0	0	0	0	0	0	1	1	0	0	0	1	0	1	1	0	0
LHUB_CNRS23599	121	t1994	publicST121	1	1	0	0	0	0	0	0	1	1	0	0	0	1	0	1	1	0	0
LHUB_CNRS23624	121	t1994	publicST121	1	1	0	0	0	0	0	0	1	1	0	0	0	1	0	1	1	0	0
LHUB_CNRS23640	121	t1994	publicST121	1	1	0	0	0	0	0	0	1	1	0	0	0	1	0	1	1	0	0
LHUB_CNRS23645	121	t21368	publicST121	1	1	0	0	0	0	0	0	1	1	0	0	0	1	0	1	1	0	0
LHUB_CNRS23679	121	t162	publicST121	1	1	0	0	0	0	0	0	1	1	0	0	0	1	0	1	1	0	0
LHUB_CNRS23696	45	t550	publicST45	1	0	0	0	0	0	0	0	1	0	0	1	1	1	1	0	0	0	0
LHUB_CNRS23734	121	t2524	publicST121	1	1	0	0	0	0	0	0	1	1	0	0	0	1	0	1	1	0	0
LHUB_CNRS23735	121	t7065	publicST121	1	1	0	0	0	0	0	0	1	1	0	0	0	1	0	1	1	0	0
LHUB_CNRS23739	121	t1994	publicST121	1	1	0	1	0	0	0	0	1	0	0	0	0	1	0	1	1	0	0
LHUB_CNRS23786	121	t2524	publicST121	1	1	0	0	0	0	0	0	1	1	0	0	0	1	0	1	1	0	0
LHUB_CNRS23791	121	t1994	publicST121	1	1	0	0	0	0	0	0	1	1	0	0	0	1	0	1	1	0	0

1: presence, 0: absence

Table S3: resistome and virulome of 33 methicillin-susceptible *Staphylococcus aureus* isolates from Belgium from October 2021 to September 2023 as well as 21 reference genomes of ST121 MSSA (Zhou et al.) [13], and 1 ST123 MSSA belonging to the European epidemic fusidic acid-resistant impetigo clone from Belgium, 2020 (Deplano et al.) [4].

This supplementary material is hosted by Eurosurveillance as supporting information alongside the article "Emergence and spread of a mupirocin-resistant variant of the European epidemic fusidic acid-resistant impetigo clone of *Staphylococcus aureus* . Belgium, 2013 to 2023", on behalf of the authors, who remain responsible for the accuracy and appropriateness of the content. The same standards for ethics, copyright, attributions and permissions as for the article apply. Supplements are not edited by Eurosurveillance and the journal is not responsible for the maintenance of any links or email addresses provided therein.
